# Evaluation of “Bias Against Disconfirmatory Evidence” in patients with functional movement disorders

**DOI:** 10.1192/j.eurpsy.2025.317

**Published:** 2025-08-26

**Authors:** I. Esteban-Avendaño, J. Torres Cortés, C. Moreno López, C. Loeck de la Puerta, A. Alonso Cánovas, I. Pareés Moreno

**Affiliations:** 1 Servicio de Psiquiatría; 2 Servicio de Neurología, Hospital Universitario Ramón y Cajal, Madrid, Spain

## Abstract

**Introduction:**

Functional neurological disorders (FND) are defined as neurological symptoms that are inconsistent and incongruent with classic neurological disorders. Over the past two decades, an interest in the potential underlying mechanisms of these disorders has occurred and a new pathophysiological framework based on current neurobiological theories about global brain function such as the predictive coding theory has emerged. Within this framework, abnormal or erroneous beliefs about symptoms, mediated by attention, are hypothesized to modulate perception and movements, ultimately leading to FND. Previous studies have evaluated cognitive biases such as the jumping to conclusion reasoning style in patients with functional movement disorders (FMD) and it has been suggested that they may play a role in symptoms production. In this study, we evaluated the behavior of patients with FMD when confronted with evidence that contradicts their beliefs through the “Bias Against Disconfirmatory Evidence” (BADE) and their tendency to accept implausible interpretations through the “Liberal Acceptance Bias” (LA).

**Objectives:**

To evaluate whether patients with FMD have greater difficulty integrating information based on disconfirming evidence than the general population.

**Methods:**

Observational case-control study in which the presence of BADE and LA biases were assessed in a sample matched by sex and age using Woodward’s BADE task. Clinical and demographic characteristics of the participants were recorded (such as “Mini Mental State Examination” (MMSE) or “Peters et al. Delusions Inventory” (PDI -21), level of education, employment situation and marital status and cohabitation situation). The BADE test analyses the scores that the patient provides at 3 points in time on the plausibility of 24 scenarios after increasing the information received.

**Results:**

Twenty patients (median age 50.5 years, 75% female) and twenty people from the control group (median age 52.50 years, 75% female) were included. No differences were found on demographic features, MMSE or PDI -21 scores. When compared to healthy controls, FMD patients scored significantly lower in BADE (median 3.35, p=0.03) and significantly higher in LA (median 3.08, p=0.017). Also, when the maximum information was provided, patients scored significantly higher in implausible situations (p=0.01) and lower in true situations (p=0.02) than the control group.

**Image 1:**

**Figure:**
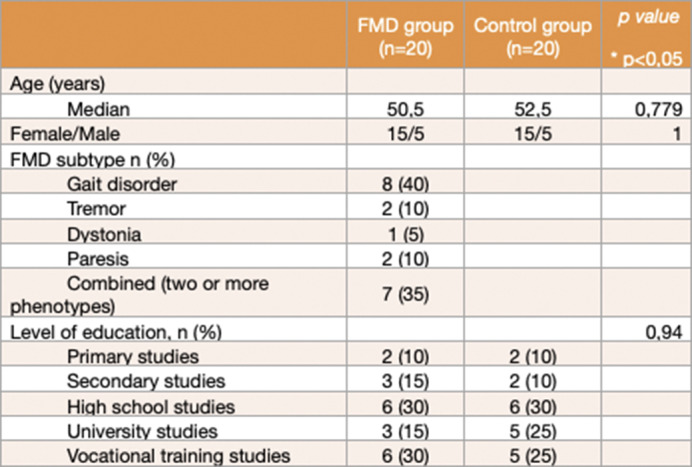

**Image 2:**

**Figure:**
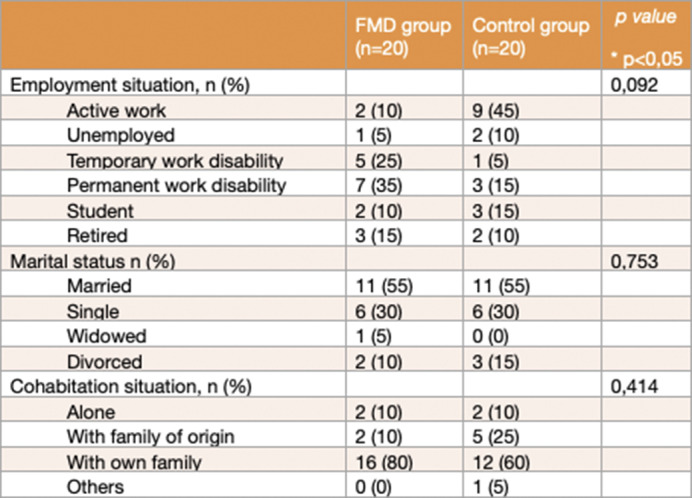

**Image 3:**

**Figure:**
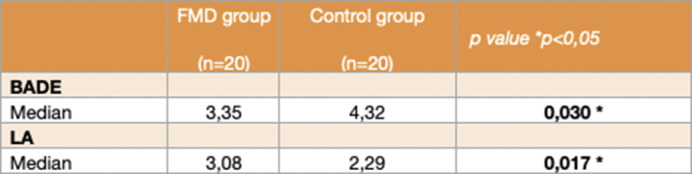

**Conclusions:**

Patients with FMD have greater difficulty in modifying their beliefs when confronted with disconfirming evidence and a greater tendency to accept less plausible options. These cognitive biases, among other factors, may facilitate the adoption of fixed beliefs, regardless of their plausibility, early and with little evidence. Our results may also explain why some patients with FMD remain with erroneous beliefs despite the explanation of the diagnosis.

**Disclosure of Interest:**

None Declared

